# IL-6在非小细胞肺癌患者筛查与预后中的研究进展

**DOI:** 10.3779/j.issn.1009-3419.2025.101.18

**Published:** 2025-10-20

**Authors:** Dongyang XU, Chenxiao QIAO, Xiaoying ZHANG, Qingchao KONG, Xiang JI

**Affiliations:** 250014 济南，山东第一医科大学第一附属医院（山东省千佛山医院）呼吸与危重症医学科; Department of Respiratory, The First Affiliated Hospital of Shandong First Medical University and Shandong Provincial Qianfoshan Hospital, Jinan 250014, China

**Keywords:** 肺肿瘤, 白细胞介素-6, 预后, 筛查, Lung neoplasms, Interleukin-6, Prognosis, Screening

## Abstract

非小细胞肺癌（non-small cell lung cancer, NSCLC）作为肺癌的主要病理类型，其筛查与预后评估对于临床治疗方案的制定和患者生存率的提升具有重要意义，并且其发生和发展与炎症反应密切相关。白细胞介素-6（interleukin-6, IL-6）作为重要的炎症介质，在NSCLC的肿瘤微环境、免疫逃逸、肿瘤进展及治疗抵抗中发挥着关键作用。近年来的研究表明，IL-6不仅参与NSCLC的生物学行为，还可能作为潜在的筛查和预后生物标志物。本文综述了IL-6在NSCLC的筛查、治疗中可能出现的不良事件以及在NSCLC相关合并症中的应用。

## Abstract (en)

Non-small cell lung cancer (NSCLC), the predominant histological subtype of lung cancer, mandates precise screening and accurate prognostic assessment to guide therapeutic strategies and improve patient survival. The initiation and progression of NSCLC are intimately linked to inflammatory responses. Interleukin-6 (IL-6), a pivotal inflammatory mediator, critically modulates the tumor microenvironment, immune evasion, tumor progression, and therapy resistance in NSCLC. Recent evidence indicates that IL-6 not only participates in the biological behavior of NSCLC but also holds promise as a potential biomarker for early detection and prognostication. This review summarizes the role of IL-6 in NSCLC screening, treatment-related adverse events, and its utility in the management of NSCLC-associated comorbidities.

肺癌在癌症中发病率最高，也是癌症相关死亡的主要原因^[1]^。非小细胞肺癌（non-small cell lung cancer, NSCLC）占所有肺癌的85%，且缺乏特异的生物标志物，大多数患者发现已处于晚期，治疗难度增加，且预后不良^[2]^。据报道^[3]^，NSCLC患者的5年生存率仅为26.4%。虽然靶向治疗与免疫治疗的兴起给NSCLC患者的治疗带来了新的希望，大多数患者并未从中受益，或未能获得持久的治疗效果^[4]^。因此迫切需要寻找能够预测NSCLC患者预后的生物标志物，为患者提供个性化治疗，提高NSCLC患者的生存率和生活质量。

慢性炎症会释放多种炎症介质如细胞因子、趋化因子等。这些介质中包含促炎因子，如白细胞介素-6（interleukin-6, IL-6）、肿瘤坏死因子-α（tumor necrosis factor-α, TNF-α）等，以及抗炎因子如IL-10、IL-13等。炎症过程中释放的血管内皮生长因子（vascular endothelial growth factor, VEGF）会促进新生血管的生成，促进肿瘤的生长，转化生长因子-β（transforming growth factor-β, TGF-β）会促进肿瘤的转移^[5]^。长期的炎症反应还会导致免疫系统功能异常，使肿瘤细胞出现免疫抑制现象，从而逃避免疫系统的清除^[6]^。许多肿瘤的发生发展都与慢性炎症有关，例如，吸入细颗粒物、烟草烟雾和石棉会导致肺部和气道的炎症，并促进肺癌和间皮瘤的发生发展^[7,8]^。总之炎症与癌症是相互促进的关系，慢性炎症会增加癌症的发生风险，癌症也会引发炎症^[6]^。研究^[9]^表明，肿瘤及肿瘤相关细胞分泌的IL-6不仅可以促进肿瘤细胞的生长，而且可以通过激活下游通路来正反馈促进IL-6的分泌。因此IL-6还作为一种有利于肿瘤生长的细胞因子，在NSCLC中得到了广泛的研究。

## 1 IL-6与NSCLC

### 1.1 IL-6相关分子信号通路

IL-6的信号通路主要包括经典信号通路[糖蛋白130（glycoprotein 130, gp130）介导的信号通路]、反式信号通路以及其他信号通路[如丝裂原活化蛋白激酶（mitogen-activated protein kinase, MAPK）信号通路和磷脂酰肌醇3-激酶/蛋白激酶B（phosphatidylinositol 3-kinase/protein kinase B, PI3K/Akt）信号通路]，3条信号通路都会促进肿瘤的进展。在经典信号通路中，IL-6首先与细胞表面的IL-6受体（IL-6 receptor, IL-6R）结合，形成IL-6/IL-6R复合物，随后该复合物与gp130结合，激活Janus激酶（Janus kinase, JAK）/信号转导和转录激活因子（signal transducer and activator of transcription, STAT）信号通路。反式信号通路中，IL-6与可溶性IL-6R（soluble IL-6 receptor, sIL-6R）结合形成复合物，再与不表达膜结合型IL-6R（membrane-bound IL-6 receptor, mIL-6R）但表达gp130的细胞结合，激活JAK2/STAT3信号通路^[10]^。此外，IL-6信号还可通过激活MAPK信号通路促进细胞的增殖和分化，通过PI3K/Akt信号通路调节细胞生长、代谢和存活等^[11,12]^。

### 1.2 IL-6在NSCLC发展过程中的双重作用

IL-6是一种多效性的细胞因子，在人体生理和病理过程中扮演着复杂且核心的角色。当机体受到损伤或感染时，IL-6会被释放。在急性炎症反应中，IL-6具有清除病原体、促进效应T细胞产生、刺激B细胞产生抗体的功能。然而在慢性炎症中，IL-6会表现出不利于人体的一面，如促进类风湿关节炎的发生发展等^[13]^。

IL-6在NSCLC中发挥着双重效应。在NSCLC发生初期，IL-6通过维持肺组织稳态和激活细胞毒性CD8^+^ T细胞发挥抗肿瘤作用，促进溶细胞分子释放并增强免疫监视功能。然而，随着肿瘤进展，IL-6逐渐转向促肿瘤效应，通过激活核因子κB（nuclear factor κB, NF-κB）信号轴促进肿瘤增殖信号的产生，并诱导髓源性抑制细胞（myeloid-derived suppressor cells, MDSCs）和M2型巨噬细胞等免疫抑制亚群的扩增，建立免疫抑制性微环境^[14,15]^。

肺磨玻璃结节（ground-glass opacity, GGO）是肺腺癌（lung adenocarcinoma, LUAD）的早期表现，分析GGO特征原发性LUAD患者手术标本发现，GGO组织中IL-6的减少会伴随着自然杀伤（natural killer, NK）细胞的减少，在GGO肿瘤微环境（tumor microenvironment, TME）中增加IL-6可以刺激NK细胞的增殖或活化^[16]^。

综上所述，IL-6的双面性本质是肿瘤与免疫系统动态博弈的缩影，其矛盾性揭示了TME信号网络的可塑性，而非分子本身的二元对立。未来研究需聚焦于界定IL-6功能转向的关键节点和TME标志物，以实现治疗策略的时空精准化。

### 1.3 IL-6在LUAD中的作用

IL-6在LUAD中的研究主要聚焦于靶向突变方面，因此本文主要介绍IL-6在靶向突变型LUAD中的作用。如图1，IL-6在Kirsten大鼠肉瘤病毒癌基因（Kirsten rat sarcoma viral oncogene, *KRAS*）突变型LUAD中，KRAS会促进IL-6的产生并增强其作用^[17-19]^。在间变性淋巴瘤激酶（anaplastic lymphoma kinase, *ALK*）突变型LUAD中，IL-6通过促进“免疫冷”TME的形成，成为治疗抵抗的关键介质^[20,21]^。在表皮生长因子受体（epidermal growth factor receptor, *EGFR*）突变型LUAD中，IL-6通过多重机制参与EGFR信号通路的调控及耐药的形成^[22-24]^。

**图1:**
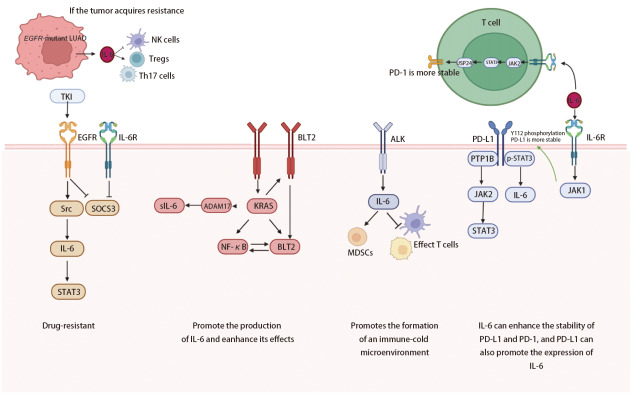
IL-6与PD-1和PD-L1的关系及在肺腺癌不同靶向突变中的分子机制

#### 1.3.1 IL-6与*KRAS*突变LUAD

*KRAS*突变通过激活下游通路（如MAPK/ERK、PI3K/Akt）诱导NF-κB和STAT3的活化，进而促进IL-6的表达和分泌。例如，在*KRAS*突变肺癌中，NF-κB的激活和IL-6的释放形成正反馈循环，增强STAT3的转录活性，从而推动肿瘤细胞增殖、血管生成和转移^[17]^。此外，*KRAS*突变上调白三烯B4受体2（leukotriene B4 receptor-2, BLT2），进一步刺激IL-6产生，而抑制BLT2可显著减少IL-6水平和肿瘤形成^[18]^。IL-6还通过反式信号传导发挥作用，*KRAS*突变促进解整合素-金属蛋白酶17（a disintegrin and metalloproteinase 17, ADAM17）活化，导致IL-6R脱落生成sIL-6R，增强IL-6反式信号，通过MAPK通路维持肿瘤生长^[19]^。

#### 1.3.2 IL-6与*ALK*突变LUAD

研究^[20]^表明，ALK阳性LUAD相较于ALK阴性LUAD表现出TME的免疫抑制特征，其特征是效应T细胞减少、MDSCs增多，以及IL-6水平显著上调。且IL-6是导致ALK阳性LUAD免疫抑制TME的主要驱动因子。值得关注的是，临床前研究^[21]^证实IL-6R抑制剂可逆转TME的免疫抑制状态，将其转化为T细胞显性状态，且与免疫检查点抑制剂（immune checkpoint inhibitors, ICIs）联合使用时产生显著协同抗肿瘤效应，这为克服*ALK*突变LUAD的免疫治疗耐药提供了新型联合策略。

#### 1.3.3 IL-6与*EGFR*突变LUAD

在NSCLC中，IL-6通过多重机制参与EGFR信号通路的调控及耐药形成。研究^[22]^表明，当EGFR通路被酪氨酸激酶抑制剂（tyrosine kinase inhibitors, TKIs）抑制时，细胞通过激活Src/IL-6/STAT3旁路途径维持存活。研究发现埃克替尼处理可同时诱导IL-6分泌和Src激活，进而磷酸化STAT3，而使用IL-6中和抗体或Src抑制剂可逆转这一效应。此外在正常细胞中，IL-6激活STAT3的这一过程会被细胞因子信号转导抑制因子3（suppressor of cytokine signaling 3, SOCS3）迅速终止。然而，IL-6R与 EGFR的结合可以使STAT3生成不受SOCS3的影响，从而对肿瘤的发生发展起到促进作用^[23]^。

在TME层面，获得性耐药肿瘤表现出更强的间质化特征和IL-6分泌能力。IL-6会抑制NK细胞功能，同时促进调节性T细胞（regulatory T cells, Tregs）和辅助性T（T helper, Th）细胞17扩增，营造免疫抑制TME^[24]^。

### 1.4 IL-6在肺鳞癌（lung squamous cell carcinoma, LUSC）中的作用

在LUSC中，IL-6的作用主要体现在慢性炎症驱动和遗传易感性两个方面。对于吸烟者，烟草暴露诱导的持续性肺部炎症是IL-6水平升高并推动癌变的途径。然而，IL-6在无吸烟史的LUSC患者中也发挥着重要作用。研究^[25]^发现，在从不吸烟的LUSC患者中，IL-6/JAK/STAT通路的表达显著增加。*IL-6*启动子变异（如rs1800797）会增加LUSC的发病风险，使患者对氡这一致癌物更加敏感。该启动子变异同样也会增加肺癌的发病风险，尤其在不吸烟患者中风险更高^[26]^。

## 2 IL-6协助NSCLC的早期筛查

在肺癌中，IL-6主要由肿瘤细胞、肿瘤相关免疫细胞（如巨噬细胞、MDSCs）产生。研究^[10]^表明，肿瘤细胞可以分泌IL-6来促进自身的生长和存活，同时也通过诱导周围免疫细胞的活化，进一步增强TME的炎症状态。例如，IL-6能够促进巨噬细胞向M2型转化，从而促进肿瘤的进展和转移。此外，MDSCs作为TME中的重要免疫抑制细胞，其分泌的IL-6不仅能够抑制抗肿瘤免疫反应，还能促进肿瘤细胞的转移和生长。因此，体内IL-6水平的升高要警惕肺癌的发生。

研究^[27,28]^表明血液中IL-6水平的升高会增加肺癌发生的风险。虽然单一的IL-6在NSCLC患者与健康人中展现出对NSCLC一定的预测作用，但是效果弱于与其他指标联合应用，如IL-6与IL-8和癌胚抗原（carcinoembryonic antigen, CEA）联合预测NSCLC的受试者工作特征（receiver operating characteristic, ROC）曲线下面积（area under the curve, AUC）值为0.883，优于IL-6单一的预测效果，AUC值为0.817^[29]^。在根据体重指数、吸烟状况、CEA和中性粒细胞-淋巴细胞比值（neutrophil-to-lymphocyte ratio, NLR）建立的预测模型中加入IL-6与IL-1受体拮抗剂（IL-1 receptor antagonist, IL-1RA）后，将模型的预测效果从最初的AUC值0.716显著提高到0.851^[28]^。

体内IL-6水平的增高不仅预示着肿瘤的发生，也表明体内存在炎症反应，因此区分IL-6升高的原因是必要的。分析诊断为NSCLC患者与肺部良性疾病（弥漫性间质性肺炎、阻塞性肺病、慢性咳嗽）患者血液IL-6水平发现，二者之间存在显著差异，肺癌患者的血液IL-6水平显著高于肺部良性疾病患者（*P*<0.001），在支气管肺泡灌洗液（bronchoalveolar lavage fluid, BALF）中也观察到了同样的结果。并且比较血液与BALF中的细胞因子水平发现，血液中IL-6水平与BALF中IL-6水平的相关性最高（*r*=0.774, *P*<0.001）^[30]^。进一步分析NSCLC患者与肺部良性疾病患者BALF中的IL-6水平发现，IL-6预测NSCLC的AUC值为0.620，敏感性为73%，特异性为69%^[31]^。

## 3 IL-6与免疫治疗疗效

多项研究^[32-35]^均观察到较高的血液IL-6水平与接受ICIs治疗的NSCLC患者的不良预后有关。免疫治疗（单药治疗与免疫联合化疗）前IL-6水平较高的NSCLC患者的无进展生存期（progression-free survival, PFS）与总生存期（overall survival, OS）更短，基线IL-6>6 pg/mL可区分获益人群。同时在治疗过程中动态监测IL-6水平发现，治疗后12周较基线水平增加>69.36%的患者的OS更短^[34]^。且免疫化疗4个周期后IL-6水平>6.58 pg/mL的患者的PFS更低。在基线、免疫化疗2个周期后和免疫化疗4个周期后，部分缓解（partial response, PR）或疾病稳定（stable disease, SD）的患者IL-6水平持续下降，疾病进展（progressive disease, PD）患者IL-6水平持续上升^[36]^。

同时，肿瘤组织中IL-6表达水平与免疫细胞的关系研究^[24,37]^发现，IL-6水平表达较高的肿瘤组织中免疫抑制细胞如MDSCs、M2型巨噬细胞和Tregs的数量较多，免疫杀伤细胞如NK细胞的数量较少，这可能是造成免疫治疗效果不佳的原因之一。有研究^[37,38]^表明IL-6的过度合成及持续表达会导致病理影响，检测肿瘤组织中的IL-6水平发现，IL-6水平较高的NSCLC患者在接受ICIs治疗后，PFS较短，并且与肿瘤原发灶-淋巴结-转移（tumor-node-metastasis, TNM）分期呈正相关。这可能与IL-6通过激活下游信号通路，促进肿瘤的转移、生长有关^[39]^。为了提高预测的准确性，IL-6与其他炎症因子联合使用也是一个很好的选择，通过IL-6和IL-15状态的结合将患者的生存结局（PFS和OS）分为3个不同的组，预测接受ICIs治疗的NSCLC患者的预后效果要优于单一指标^[33]^。

在分子层面，如图1，IL-6不仅会增强程序性死亡配体1（programmed death-ligand 1, PD-L1）和程序性死亡受体1（programmed death-1, PD-1）的稳定性，PD-L1也会促进IL-6的表达。研究^[40]^发现PD-L1高表达的NSCLC细胞通过结合并抑制蛋白酪氨酸磷酸酶1B（protein tyrosine phosphatase 1B, PTP1B）来激活JAK2/STAT3信号通路，同时PD-L1还与核内的磷酸化STAT3（phosphorylated-STAT3, p-STAT3）结合，增强其转录活性，促进IL-6等细胞因子的表达。这些细胞因子进一步促进MDSCs的招募并增强其免疫抑制功能，从而抑制抗肿瘤免疫反应。并且，生信分析^[41]^也证实PD-L1的高表达会促进IL-6/JAK2/STAT3信号通路的活化。相反，IL-6也会激活JAK1，进一步磷酸化PD-L1的Y112位点，后者会招募N-糖基转移酶STT3A，进而催化PD-L1的糖基化，增强其稳定性^[42]^。IL-6还会通过JAK2/STAT3通路激活泛素特异性肽酶24（ubiquitin-specific peptidase 24, USP24）增强PD-1蛋白稳定性从而抑制T细胞的抗肿瘤反应^[43]^。

综上所述，IL-6无论是在临床研究还是分子机制层面都与ICIs治疗有着极大的关系，因此IL-6有望成为ICIs治疗的潜在预后标志物。

## 4 IL-6与放化疗疗效

放疗和化疗，不仅会损害肿瘤细胞，也会对周围的正常组织造成损伤。损伤的组织会释放IL-6、TNF-α等细胞因子来应对损伤。但过度或持续的炎症反应可能会对抗肿瘤治疗效果产生负面影响。此外，放化疗主要通过破坏肿瘤细胞的DNA来达到抗肿瘤的效果，而在放化疗过程中产生的IL-6会促进肿瘤细胞的DNA修复，这会使放化疗耐药情况的出现^[44,45]^。由此可见，IL-6在放化疗抵抗中起着关键作用，因此监测体内IL-6水平有利于判断疗效。

分析328例接受根治性同步放化疗与贯序放化疗的NSCLC患者发现，IL-6水平增高会增加1年内死亡的风险（OR=2.17, *P*=0.005）。根据IL-6、可溶性PD-L1（soluble PD-L1, sPD-L1）、干扰素γ诱导蛋白10（interferon-γ-inducible protein 10, IP-10）绘制列线图对1年内死亡的风险起到了良好的预测作用[AUC=0.774 (95%CI: 0.716-0.832)]，在39例的验证队列中AUC=0.734（95%CI: 0.565-0.902）^[46]^。另一项研究^[47]^纳入75例接受根治性胸部放疗的NSCLC患者，发现与治疗后IL-6降低的患者相比，治疗后IL-6升高的患者OS（*P*=0.013）和PFS（*P*=0.025）显著缩短。

在放疗过程中，肺组织受到辐射损伤后，IL-6的表达水平会显著上升，这一过程会消耗氧气导致肺组织缺氧，缺氧会促进活性氧（reactive oxygen species, ROS）的产生，进一步上调TGF-β，促进胶原蛋白的形成，从而降低肺泡的弹性，最终引发放射性肺炎（radiation pneumonitis, RP）^[48]^。

研究^[49,50]^表明IL-6能够预测RP的发生风险。针对105例接受过放疗的肺癌、食管癌、纵隔肿瘤患者的研究^[49]^发现，RP患者和非RP患者的IL-6基线水平存在显著差异（*P*<0.05）；并且RP患者的IL-6水平在2周内开始升高，早于确认RP可疑症状而进行的常规计算机断层扫描（computed tomography, CT）检查（4周）。根据IL-6与C反应蛋白（C-reactive protein, CRP）绘制的ROC曲线对RP预测的AUC值为0.92（95%CI: 0.83-0.97）。IL-6还会增加RP的严重程度，分析15例发生RP的NSCLC患者的研究^[50]^发现，在治疗第3周时RP≥2级患者的IL-6水平与RP<2级的患者存在显著差异（*P*=0.05）。

## 5 IL-6与靶向治疗

IL-6会造成NSCLC对TKIs的耐药，这可能与IL-6会诱导上皮间充质转化（epithelial-mesenchymal transition, EMT）从而增强肿瘤的侵袭性有关，在动物实验中也证实抑制IL-6可逆转TKIs耐药^[24]^。IL-6在靶向治疗中的研究多集中于*EGFR*突变的患者，分别研究70例接受奥希替尼治疗和226例接受吉非替尼治疗的NSCLC患者发现，基线IL-6≥7 mg/L的患者有着较差的PFS，基线IL-6≥7 mg/L是PFS较差的独立预测因子（吉非替尼：HR=2.09，*P*=0.009；奥希替尼：HR=2.09，*P*=0.009），并且耐药时IL-6水平较基线水平显著增高（吉非替尼：*P*=0.019；奥希替尼：*P*=0.002）^[51]^。Sheng等^[52]^研究54例接受吉非替尼治疗的NSCLC患者发现，治疗有效的患者IL-6水平较基线时显著降低（*P*<0.001）。Jia等^[53]^研究发现基线IL-6水平较低的NSCLC患者接受吉非替尼治疗后有着更好的客观缓解率（objective response rate, ORR）。

有研究^[54]^发现，IL-6的Asn73位点发生N-糖基化修饰时会激活JAK-STAT3信号通路，促进细胞增殖。而N-糖基化缺陷IL6（defective N-glycosylation interleukin-6, deNG-IL6）会激活Src-Yes相关蛋白（Yes-associated protein, YAP）-SRY盒转录因子2（SRY-box transcription factor 2, SOX2）信号轴促进肺癌细胞的转移。在奥希替尼耐药的肺癌细胞中，deNG-IL6的分泌增加，这可能是奥希替尼耐药的机制之一。因此监测deNG-IL6水平有助于对奥希替尼疗效作出判断，并且deNG-IL6也有望成为逆转奥希替尼耐药的新靶点。

综上所述，IL-6在TKIs耐药方面起着重要作用，因此，监测体内IL-6水平有助于对疗效作出判断。总之，IL-6 的预测价值已在奥希替尼/吉非替尼治疗的*EGFR*突变患者中得到验证；而其他血液指标如NLR、PLR等，则对厄洛替尼治疗的*EGFR*突变患者及克唑替尼治疗的*ALK*突变患者的疗效具有预测意义^[55,56]^。

## 6 IL-6与NSCLC治疗相关并发症及合并症

### 6.1 IL-6与免疫相关不良事件（immune-related adverse events, irAEs）

ICIs虽然为肺癌患者带来了更高的生存获益，但有时会引起一系列的irAEs，分析221例接受ICIs治疗的NSCLC患者发现基线水平及治疗过程中的IL-6水平并不能预测irAEs的发生^[57]^。而另一项纳入41例接受ICIs治疗的NSCLC患者（21例有恶病质）的研究^[58]^发现，基线和治疗期间较高的IL-6会增加irAEs的风险，并且患有恶病质的NSCLC患者较不患有恶病质的相比，体内IL-6水平更高。这提示恶病质可能是导致两项研究结果差异的混杂因素；因此，难以明确irAEs风险的增加究竟是由IL-6本身还是由恶病质状态所驱动。

关于19例无慢性阻塞性肺疾病（chronic obstructive pulmonary disease, COPD）患者和80例合并不同程度COPD的NSCLC患者的研究^[59]^发现，重度COPD患者的irAEs发生率要显著高于无COPD以及轻中度COPD的患者（*P*=0.003），这说明irAEs的发生受患者身体情况的影响较大，研究时需要注意区分不同的人群。

ICIs相关肺炎（ICIs-related pneumonitis, CIP）是ICIs治疗中常见的不良反应。一项荟萃分析^[60]^显示，肺癌比其他癌症更有可能导致所有级别或高级别CIP。IL-6作为一种促炎因子，具有炎症放大作用，在肺部会造成组织损伤，因此IL-6的升高要警惕肺炎的发生，并且IL-6的升高可能会提示肺炎程度的加重。

关于87例接受ICIs治疗的NSCLC患者的研究^[61]^发现，发生CIP时IL-6水平与基线时存在显著差异（*P*=0.001），而在87例接受ICIs治疗的未发生CIP的对照组NSCLC患者中，基线IL-6水平较最后一次接受ICIs治疗前IL-6水平显著下降（*P*=0.030）。这表明IL-6的检测对辅助诊断CIP具有重要意义。并且在发生CIP的患者中，高IL-6水平的患者OS更短。对45例发生CIP的NSCLC患者进行ICIs再激发发现，CIP时较高的IL-6水平（25 *vs* 7.6 pg/mL）更容易发生复发性肺炎（*P*=0.007）；并且IL-6是影响初始CIP患者OS的独立危险因素（HR=0.06, *P*=0.015）^[62]^。术后第1天的CRP、IL-6、胰岛素样生长因子-1（insulin-like growth factor 1, IGF-1）联合预测老年肺癌患者（>70岁）术后肺部感染AUC值为0.871，敏感性为0.835，特异性为0.821；并且术后第1天高水平的IL-6会增加感染风险（OR=1.064, *P*<0.001）^[63]^。

### 6.2 IL-6与心率变异性（heart rate variability, HRV）

HRV是自主神经系统功能和心血管健康的重要指标，反映了心脏适应各种生理和病理条件的能力^[64]^。对131例NSCLC的患者的研究^[65]^发现，基线IL-6>6.74 pg/mL是HRV下降的独立预测因子（OR=3.203, *P*=0.033），HRV的下降往往预示着自主神经功能障碍，会增加癌症的死亡风险。IL-6联合静息心率（resting heart rate, RHR）、血清钠建立的风险评分对诊断HRV的AUC值为0.849，敏感性为76.39%，特异度为79.66%。在43例NSCLC患者的验证队列中的AUC为0.788，并且该评分对NSCLC患者的OS（未说明治疗方法）也展现出了良好的预测效能（*P*<0.01）。

### 6.3 IL-6与NSCLC转移

EMT是肺癌转移的主要机制之一，而IL-6会促进这一过程。对120例无脑转移的NSCLC患者按IL-6水平分为两组并随访28个月后发现，随访前IL-6水平更高的患者脑转移发生率更高（35% *vs* 17%, *P*=0.012）。在细胞层面的研究^[66]^发现，脑转移倾向的NSCLC细胞（A549-F3）能分泌大量IL-6，IL-6激活小胶质细胞的JAK2/STAT3信号通路，诱导其向M2表型极化。M2型小胶质细胞能促进NSCLC细胞在大脑中的定植和生长。

在骨转移方面，IL-6会增强肺癌细胞的迁移能力和骨破坏能力^[67]^。一项纳入127例发生骨转移与78例无骨转移的肺癌患者的研究^[68]^发现，IL-6虽然对骨转移的发生率展现出一定效果（AUC=0.65），但是效果弱于其他细胞因子如骨保护素（osteoprotegerin, OPG）（AUC=0.83）、甲状旁腺激素相关肽（parathyroid hormone-related peptide, PTHrP）（AUC=0.83）、骨转移标志物总I型前胶原N端前肽（total procollagen type I N-terminal propeptide, tP1NP）（AUC=0.83）和β-I型胶原交联C端肽（β-crosslinked telopeptide of type I collagen, β-CTx）（AUC=0.83）。肿瘤细胞的侵袭性增强，也是肿瘤耐药的原因之一，因此转移风险的增加也提示着肿瘤的治疗效果不佳。

## 7 IL-6抑制剂在NSCLC的临床试验进展

### 7.1 IL-6抑制剂疗效差异与患者选择的关键作用

在CANOPY-1实验^[69]^中，针对IL-6上游信号IL-β的卡那单抗联合帕博利珠单抗，相较于安慰剂联合帕博利珠单抗，并未观察到患者的OS获益（20.8 *vs* 20.2个月），在亚组分析中也并未发现卡那单抗联合帕博利珠单抗在基线水平CRP及IL-6较高的患者中疗效更佳。而一项针对恶病质、CRP≥50 mg/L和IL-6浓度超过正常上限2倍的NSCLC患者的回顾性研究^[70]^发现，针对IL-6的托珠单抗联合抗肿瘤治疗组相较于传统的抗肿瘤治疗组，患者的OS显著延长（15.1 *vs* 3.2个月）。两项研究结果存在差异，可能也是因为患者基线特征存在显著差异。CANOPY-1研究人群的美国东部肿瘤协作组体能状态（Eastern Cooperative Oncology Group performance status, ECOG PS）评分为0和1分，而后者研究人群的ECOG PS评分为2-4分。患者选择不当可能是关键因素，未来需进一步细化研究人群。此外，肿瘤细胞可分泌IL-6，而这一过程可能不需要IL-1β的参与。虽然IL-6能够预测治疗效果，但是并没有发现卡那单抗的有效性，这说明不能只关注IL-6水平的高低，还应结合ECOG PS评分等反映患者状态的方法来指导治疗。

### 7.2 IL-6抑制剂对irAEs的治疗作用

一项针对92例接受抗IL-6抑制剂肿瘤（肺癌、黑色素瘤、泌尿系统肿瘤）患者的回顾性分析^[71]^显示，其适应证涵盖炎症性关节炎（73%）、肝炎（7%）、肌炎/心肌炎（5%）等多种irAEs。在启动抗IL-6R治疗后，73%的患者在中位2个月内实现irAEs缓解或降级至≤1级，且仅7%患者因不良事件停药。对于既往因高级别irAEs（如4级肝炎）而中止ICIs治疗的患者，预防性联用托珠单抗可支持ICIs再挑战。例如，1例IVB期LUAD患者在帕博利珠单抗再挑战时联用托珠单抗，实现了长达4年的疾病控制，且新发irAEs（甲状腺炎、垂体炎）均可通过激素控制^[72]^。

### 7.3 IL-6抑制剂与ICIs联合应用的潜力

现有研究^[71]^表明，IL-6抑制剂在控制irAEs的同时，可能保留甚至增强ICIs的抗肿瘤疗效。在70例可评估患者中，抗IL-6R治疗前后的ORR均为66%，且完全缓解率提升8%。黑色素瘤亚组中，ORR更从56%显著提升至68%（*P*=0.04）。总之，将ICIs与IL-6抑制剂联合使用，托珠单抗调节免疫抑制，同时防止过度炎症，可能是一种双赢的策略。

## 8 IL-6的动态监测方案

综合本文，基线水平IL-6高是NSCLC治疗的不良预后因素，但是在不同研究中IL-6的预后临界值存在差异，这可能是由检测方法（检测试剂盒的来源）、临界值的选择方法（参考既往文献或基于ROC曲线确定最佳临界值）、检测样本（血浆或血清）的不同所致^[32-34]^。然而，一个更具临床价值的共性发现是：无论基线水平如何，治疗过程中IL-6水平的升高，一致性地预示了疗效不佳和疾病进展^[34,36,47]^。这表明相比于一个固定的阈值，IL-6的动态变化趋势可能是一个更可靠且不受绝对阈值困扰的预后指标。

因此我们建议在治疗前（基线）及每个治疗周期结束后定期检测IL-6水平，以动态评估疾病进展、治疗反应及并发症风险。监测频率可根据治疗类型和患者个体情况调整，但基线值和治疗周期后变化是关键参考指标。此外，IL-6对不同治疗方案疗效的预测效果总结见表1。

**表1:** IL-6对不同治疗方案的预测效果

Application scenario	Predictive effect
Immunotherapy	Measure IL-6 at baseline and after every treatment cycle, If increased, be cautious of poor treatment effect and the occurrence of CIP.
Chemoradiotherapy	Baseline to determine treatment efficacy and the risk of RP, re-examine at 2 weeks of treatment, if increased from baseline, be cautious of RP occurrence. An increase in levels after definitive thoracic radiotherapy is associated with poor prognosis.
Targeted therapy	Currently only compared IL-6 levels when the patients with EGFR mutation have poor outcomes after baseline treatment, this cannot promptly judge treatment efficacy.
NSCLC-related complications and comorbidities	The IL-6 levels at baseline and during treatment for predicting irAEs are uncertain. A baseline IL-6 >6.74 pg/mL increases the risk of HRV decrease, high levels increase the risk of brain metastasis, and the predictive effect for bone metastasis is not good.

CIP: checkpoint inhibitor-related pneumonitis; RP: radiation pneumonitis; NSCLC: non-small cell lung cancer; irAEs: immune-related adverse events; HRV: heart rate variability.

## 9 小结与展望

IL-6作为一种炎症因子，在促进肿瘤生长、转移以及耐药方面都起着重要作用，在预测乳腺癌、胃癌、肝癌的预后方面展现出了一定潜力^[73-75]^。在NSCLC的筛查与预后方面也展现出一定的作用。但是目前在临床中的应用仍面临以下挑战。

### 9.1 IL-6检测的临床挑战与标准化需求

尽管血液IL-6水平在NSCLC筛查与预后评估中展现出重要潜力，但其临床应用仍面临显著挑战。首要问题在于IL-6的生物学特性：其在血液中的半衰期较短，且存在明显的昼夜波动，在健康人中8:00和21:00时有两个最低点，在约19:00和5:00有两个最高点，这使得单次检测结果易受采样时间点影响^[76]^。但是在肿瘤患者中是否会因为慢性炎症而削弱昼夜波动有待进一步研究。目前临床上多在清晨空腹状态下采血以减少食物等因素的影响，以保证患者在相同的生理条件下检测，这也使结果更具有横向对比意义。

在检测标本的选择上，组织检测虽能直接反映TME中的IL-6表达，但其侵入性操作限制了重复动态监测的可行性。相比之下，血液检测具有无创、可及性高且便于连续采样的显著优势，更适用于疗效监测和预后动态评估。组织和血液中高水平的IL-6都会导致NSCLC的不良预后，提示检测组织水平与血液水平的IL-6在效果上可能相同^[37]^。

展望未来，推动IL-6检测的标准化是将其转化为可靠临床工具的关键。这包括：（1）规范采样流程：明确统一的采血时间（如固定于清晨），以尽量减少昼夜波动带来的变异。（2）统一检测方法：建立并推广标准化的检测平台与试剂，确保不同中心间数据的可比性。（3）定义临界值：通过大规模前瞻性研究，确定针对NSCLC筛查、预后分层及疗效预测的特异性IL-6临界值。

### 9.2 IL-6预后价值向治疗干预转化的障碍

虽然血液IL-6水平是NSCLC治疗效果不佳的预后因素，而检测IL-6水平的意义在于能够区分不同的人群、及时发现病情变化以指导治疗方案。虽然IL-6在不同治疗方法的疗效预测中都显示出了一定优势^[33,46,51]^。然而，目前尚缺乏IL-6抑制剂与常规疗法联合应用的大规模前瞻性临床研究，且IL-6抑制剂在ECOG PS评分较低患者中的治疗指导价值仍不明确。现有证据提示，患者基线特征（如ECOG PS评分）的差异可能显著影响疗效评估^[69,70]^。未来需通过更精细的人群分层研究，结合IL-6水平与患者整体状态，以优化治疗策略。

### 9.3 IL-6多指标联合与机器学习模型

肿瘤的发生发展是一个复杂的过程，受多种因素的影响，单一的指标也难以全面反映这些因素。文中也提到，IL-6与其他细胞因子或者能够反映肿瘤情况的其他指标联合应用，在筛查及预后方面的预测效果也会优于IL-6单一指标^[29,33]^。这也将是未来一个值得研究的方向。

随着机器学习的发展，利用可获取的血液指标建立机器学习模型，预测NSCLC相关的不良事件（如肿瘤转移和治疗相关并发症）的发生时间，早于患者出现相应症状后进行辅助检查的时间。如基于血液中OPG、PTHrP、tP1NP和β-CTx四种指标建立的骨转移预测模型，其AUC值达到0.940。在一项涉及44例NSCLC患者的前瞻性研究^[68]^中，该模型的预测结果与骨扫描结果高度一致，且平均预测时间比骨扫描提前9.46个月。这凸显了血液指标在预测NSCLC患者各种不良情况中的重要价值，早期预测为医生争取了宝贵的时间，以便提前干预，改善患者预后，提高生活质量。此外，IL-6在肿瘤发生和发展过程中发挥重要作用，未来有望在各种模型中扮演重要角色。
